# An Incidental Discovery of a Large Falciform Ligament Defect During Laparoscopic Sleeve Gastrectomy

**DOI:** 10.7759/cureus.68930

**Published:** 2024-09-08

**Authors:** Omar Eldurssi, Ali Albaqshi, Ahmed Attia

**Affiliations:** 1 Surgery, University of Benghazi Faculty of Medicine, Benghazi, LBY; 2 Surgery, London Hospital Kuwait, Kuwait, KWT; 3 Bariatric Surgery, Al-Sabah Hospital, Kuwait, KWT

**Keywords:** asymptomatic, bariatric, defects, falciform ligament, laparoscopic sleeve gastrectomy

## Abstract

The falciform ligament is a thin fold of peritoneum that attaches the liver to the anterior abdominal wall and the diaphragm. Its defect refers to an abnormal opening in the falciform ligament, a very rare condition that can be congenital or due to acquired causes. In this report, we present a case of a 42-year-old patient with a large asymptomatic falciform ligament defect incidentally discovered during laparoscopic sleeve gastrectomy. Due to the potential risk of small bowel herniation through the defect, we recommend cutting the remaining ligament to avoid this unwanted complication, as we did here.

## Introduction

The falciform ligament defects are very rare. In all of the medical literature, there are only a few cases [1]. They can be congenital or due to acquired causes [1,2]. This paper presents a large asymptomatic falciform ligament defect that was detected and prophylactically treated during laparoscopic sleeve gastrectomy.

## Case presentation

**Figure 1:**
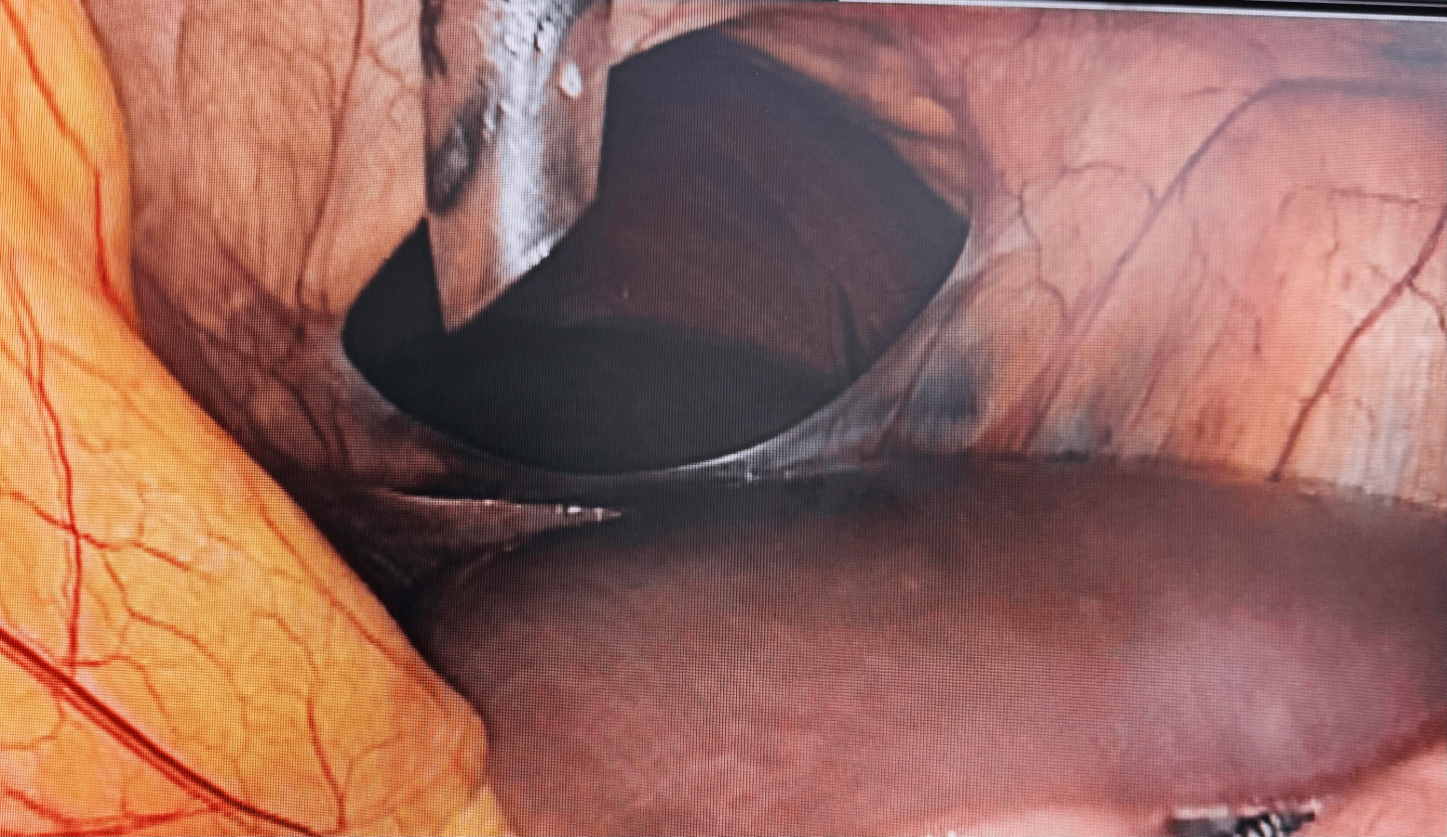
Patient's falciform ligament defect.

A 42-year-old male with a high body mass index of 40 kg/m^2^ had no comorbidities. He could not reduce his weight through lifestyle modification and had no remarkable surgical, traumatic, or medical history. Clinical examination was normal. Preoperative routine blood tests and upper gastrointestinal endoscopy were both normal (Table 1). He was scheduled for laparoscopic sleeve gastrectomy. When the camera was introduced into the abdominal cavity, we noticed a large oval defect in the falciform ligament, measuring 10x8 cm in diameter, with no organ herniation, as shown in Figure 1 and Video 1. At the end of the planned procedure, the above-mentioned defect was divided using an electrocautery device, and the operation was finalized. After the patient regained consciousness, he was informed about this additional surgical procedure. At the postoperative follow-up visits, the patient had no complaints regarding this issue. The last visit was about one month after the operation.

**Table 1:** Blood tests on admission. MCV: Mean Corpuscular Volume; MCH: Mean Corpuscular Hemoglobin; MCHC: Mean Corpuscular Hemoglobin Concentration; ALT: Alanine Aminotransferase; AST: Aspartate Aminotransferase; APTT: Activated Partial Thromboplastin Time.

Test	Value	Normal range
Erythrocytes	5.19x10^6^/uL	4.2-5.8
Hemoglobin	15.2 g/dl	13-15
Hematocrit	46.9%	39-54
MCV	90.3 Fl	82-97
MCH	29.3 pg	27-33
MCHC	32.4 g/dl	31-36
Platelet	255 x 10^3^/ul	150-400
Leucocytes	4.82 x 10^3^/uL	4.0-11.0
ALT	26.8 U/L	0-30
AST	24.8 U/L	10-37
Fasting glucose	5.1 mmol/L	3.9-5.6
Creatinine	46.8 mmol/L	53-97
Sodium	147 mmol/L	135-148
Potassium	4.5 mmol/L	3.5-5
Urea	2.8 mmol/L	0-8.3
APTT	32.0 sec	28-42
APTT normal control	31.0 sec	24-32
Bleeding time	1 min 05 sec	2 minutes to 5 minutes
Clotting time	6 min 40 sec	4 min to 10 min

**Video 1:** Intraoperative record showing the patient’s falciform ligament defect.

## Discussion

The falciform ligament morphologically divides the liver into right and left lobes. It is a peritoneal fold connecting the liver to the anterior abdominal wall and is derived from the ventral mesentery during fetal development. It also houses the umbilical vein, which changes into the ligamentum teres after birth [3]. Defects in this ligament are thought to be caused by the umbilical vein or its branches not fully closing or regressing, leaving a patent hole [4]. Alternatively, the integrity of the ligament may be compromised by neoplasia, trauma, surgery, infection, or inflammation. The etiology may be complex and often remains obscure. Falciform ligament anomalies are exceptionally rare, with scant case reports in the literature. In a study by Sato S et al. [5], a partial defect of this ligament was observed in 0.3% of the 1802 consecutive patients who underwent laparoscopic procedures from 1981 to 1994 in Japan. The anomaly can be asymptomatic, discovered incidentally during imaging or surgery, or present with acute or chronic symptoms [2,6]. Its associations with other congenital or acquired anomalies, such as umbilical hernia, omphalocele, diaphragmatic hernia, Meckel’s diverticulum, and others, have been reported but lack consistency [7,8,9,10]. The anomaly can be categorized into two main types based on defect location: supraumbilical and infraumbilical. Supraumbilical defects, more prevalent, occur in the upper part of the ligament near the diaphragm or liver, while infraumbilical defects manifest in the lower part near the umbilicus or abdominal wall. Furthermore, four patterns, slit-like, round, oval, and irregular, describe the shape and size of the defects, providing additional insights into anatomical variations. Clinical manifestations vary, encompassing abdominal pain, distension, palpable masses, nausea, vomiting, constipation, diarrhea, jaundice, fever, and hematemesis. Complications, including obstruction, ischemia, perforation, infection, hemorrhage, and strangulation, pose serious risks, potentially leading to peritonitis, sepsis, shock, or death [11]. Obstruction and ischemia are the most common complications, affecting various intra-abdominal organs. Diagnostic approaches involve a thorough clinical history, physical examination, and imaging studies. Imaging modalities such as plain radiography, ultrasonography, CT, and MRI aid in confirming the presence and location of the defect, identifying herniated contents, and detecting complications. Conservative approaches involve observation in asymptomatic cases. Surgical options include open or laparoscopic repair if internal hernia or abscess formation occurs. Some authorities, as we did in this case, recommend treating incidentally intraoperatively detected falciform ligament defects to prevent future unwanted morbidities and/or mortalities [2,11,12]. Because this simple measure of dividing the remaining ligament does not affect the fate of the planned surgical procedure, we recommend this approach as well.

## Conclusions

The falciform ligament defect represents a rare condition with a diverse clinical spectrum and potential complications. Most asymptomatic defects are discovered laparoscopically for other reasons. Diagnosis and management require consideration of various factors, including defect size, location, herniated content, and the presence of complications. For asymptomatic defects found incidentally, we recommend dividing the remnant of the ligament to prevent future internal hernias. However, further detailed studies are needed on this issue.
